# Biochemical analyses of a novel acidophilic GH5 β-mannanase from *Trichoderma asperellum* ND-1 and its application in mannooligosaccharides production from galactomannans

**DOI:** 10.3389/fmicb.2023.1191553

**Published:** 2023-06-09

**Authors:** Fengzhen Zheng, Abdul Basit, Jiaqiang Wang, Huan Zhuang, Jun Chen, Jianfen Zhang

**Affiliations:** ^1^College of Biological and Environmental Engineering, Zhejiang Shuren University, Hangzhou, China; ^2^Department of Microbiology, University of Jhang, Jhang, Pakistan; ^3^Department of ENT and Head and Neck Surgery, The Children's Hospital Zhejiang University School of Medicine, Hangzhou, Zhejiang, China; ^4^Interdisciplinary Research Academy, Zhejiang Shuren University, Hangzhou, China

**Keywords:** *Trichoderma asperellum*, β-mannanase, enzyme characteristics, action model, synergism

## Abstract

In this study, an acidophilic GH5 β-mannanase (TaMan5) from *Trichoderma asperellum* ND-1 was efficiently expressed in *Pichia pastoris* (a 2.0-fold increase, 67.5 ± 1.95 U/mL). TaMan5 displayed the highest specificity toward locust bean gum (*K*_m_ = 1.34 mg/mL, *V*_max_ = 749.14 μmol/min/mg) at pH 4.0 and 65°C. Furthermore, TaMan5 displayed remarkable tolerance to acidic environments, retaining over 80% of its original activity at pH 3.0–5.0. The activity of TaMan5 was remarkably decreased by Cu^2+^, Mn^2+^, and SDS, while Fe^2+^/Fe^3+^ improved the enzyme activity. A thin-layer chromatography (TLC) analysis of the action model showed that TaMan5 could rapidly degrade mannan/MOS into mannobiose without mannose via hydrolysis action as well as transglycosylation. Site-directed mutagenesis results suggested that Glu^205^, Glu^313^, and Asp^357^ of TaMan5 are crucial catalytic residues, with Asp^152^ playing an auxiliary function. Additionally, TaMan5 and commercial α-galactosidase displayed a remarkable synergistic effect on the degradation of galactomannans. This study provided a novel β-mannanase with ideal characteristics and can be considered a potential candidate for the production of bioactive polysaccharide mannobiose.

## 1. Introduction

Galactomannans are the main components of hemicellulose and can be transformed into various high-value products (Barak and Mudgil, 2014; Yamabhai et al., 2016; Ponzini et al., 2019; Behera et al., 2022; Sharma et al., 2022). They are composed of repeating mannose residues linked with β-1,4-glycosidic bonds and side chains of α-1,6-linked galactose side groups (Dhawan and Kaur, 2007; Ghosh et al., 2013; Sébastien et al., 2014). Common galactomannans contain locust bean galactomannan (LBG; mannose/galactose 4:1) and guar galactomannan (GG; mannose/galactose 2:1) (Moreira and Filho, 2008; Hlalukana et al., 2021; Kumar Suryawanshi and Kango, 2021). Efficient bioconversion of galactomannan is performed by the action of glycoside hydrolase (GH), particularly endo-β-1,4-mannanase (EC 3.2.1.78), which catalyzes the random cleavage of β-1,4-mannosidic bonds to produce short mannooligosaccharides (MOS) and mannose (Chauhan et al., 2012; Liu et al., 2020; Chen et al., 2021). β-mannanases are categorized into GH families of GH5, 26, 45, 113, and 134 based on the Carbohydrate-Active EnZymes (CAZy) database (http://www.cazy.org). Moreover, GH5 mannanases are gaining worldwide interest for their industrial applications, *e.g*., food, feed, and biofuel production (Dhawan and Kaur, 2007; Do et al., 2009; Katrolia et al., 2013; Srivastava and Kapoor, 2017; Kaira and Kapoor, 2021).

Owing to the recalcitrance structure of galactomannans, effective and strong β-mannanases with high acidophilic and acid-stable are preferred for efficient degradation of galactomannans to yield prebiotic MOS, with a degree of polymerization (DP) ranging from 2 to 10 units (Gómez et al., 2017; Jana et al., 2018). MOS (particularly mannobiose) can selectively inhibit the growth of the enteropathogen and improve the growth of beneficial microorganisms such as *Lactobacillus* sp. (Pongsapipatana et al., 2016; Srivastava et al., 2017; Mao et al., 2018). Moreover, MOS also possess some beneficial functions, such as anti-cancer, antioxidant, anti-diabetic, and anti-inflammatory properties as a non-digestible food ingredient (Rungruangsaphakun and Keawsompong, 2018; Jana et al., 2021). Recent developments suggested that prebiotic MOS with a DP of <4 exhibited higher solubility and diffusion and possessed better health-improving effects compared with high DP MOS (>4) (Endo et al., 2016; Jana and Kango, 2020; Kumar Suryawanshi and Kango, 2021). Various methods have been explored for MOS production, including enzymatic hydrolysis, chemical, or the cooperation of these methods (Moreira and Filho, 2008; Li et al., 2018; Jana et al., 2021). The former method is seen as a more promising way for the production of MOS because enzymatic hydrolysis is highly efficient and eco-friendly with less pollution (Arnling Bååth et al., 2018; Hlalukana et al., 2021). In addition, galactomannans decorated by α-1,6-linked d-galactose lead to steric hindrance while connecting with enzymes (Xia et al., 2016), and thus, α-galactosidase coupled with β-mannanase will perform better and yield higher amounts of MOS (Wang et al., 2010; Malgas et al., 2015; Song et al., 2018).

To date, a variety of β-mannanases are widely distributed in different microbial sources, including fungi and bacteria (Jiang et al., 2006; Dhawan and Kaur, 2007; Dawood and Ma, 2020). β-mannanases from fungi, such as species of *Trichoderma harzianum* (Ferreira and Filho, 2004), *Rhizomucor miehei* (Katrolia et al., 2013), and *T. reesei* (Ma et al., 2018) have attracted huge attention due to their excellent characteristics and significant potential for industrial applications. Fungal β-mannanases expression can be performed by submerged fermentation, but they are generally secreted in combination with mannosidase, which is disadvantageous in terms of MOS quality (Katrolia et al., 2012; Srivastava and Kapoor, 2017). *P. pastoris* is an excellent host for the production of heterologous proteins (Looser et al., 2015). Furthermore, different strategies have been attempted to improve enzymes' production in *P. pastoris*, including codon optimization (Taylor et al., 2018; Yang and Zhang, 2018). Therefore, it is highly desirable to recommend *P. pastoris* systems for the cost-effective production of β-mannanases.

*T. asperellum* ND-1 was a newly isolated biomass degradation fungus, which can efficiently secrete multiple kinds of enzymes, including β-xylosidases and xylanases (Zheng et al., 2020, 2022). Here, a novel GH5 β-mannanase gene (*TaMan5*) from *T. asperellum* ND-1 was identified and highly expressed in *P. pastoris* through codon optimization. Pivotal catalytic residues of the β-mannanase (TaMan5) were confirmed by site-directed mutagenesis, the action model on MOS was analyzed, and the synergistic action of TaMan5 coupled with commercial α-galactosidase was further investigated on the hydrolysis of galactomannans.

## 2. Materials and methods

### 2.1. Strains and chemicals

*T. asperellum* ND-1 (GenBank no: MH496612) was selected from the soil by our group. pPICZαA vector, *P. pastoris* X-33, and *Escherichia coli* DH5α (Invitrogen, America) were applied for β-mannanase heterologous expression, respectively. *p*-Nitrophenyl (pNP)-β-d-galactopyranoside (pNPG), *p*-nitrophenyl-α-l-arabinofuranoside (pNPAf), *p*-nitrophenyl-β-d-cellobioside (pNPC), sodium carboxymethyl cellulose (CMC-Na), locust bean gum (LBG), and guar gum (GG) were purchased from Sigma-Aldrich (USA). Restriction enzyme *Sac*I and PNGase F were purchased from New England Biolabs (Ipswich, MA, USA). Mannohexaose (M6), mannopentaose (M5), mannotetraose (M4), mannotriose (M3), mannobiose (M2), and beechwood xylan were purchased from Megazyme (Wicklow, Ireland). 3,5-Dinitrosalicylic acid (DNS), d-mannose, and commercial α-galactosidase (EC 3.2.1.22, *Aspergillus niger*) were purchased from Shanghai Aladdin Biochemical Technology (Shanghai, China).

### 2.2. Cloning and sequence analyses

A full-length β-mannanase gene (*TaMan5*) was identified from the *T. asperellum* ND-1 genome sequence. The amino acid and nucleotide sequences have been deposited in the GenBank under the accession numbers UNE56028.1 and OM128445.1, respectively. Prediction of signal peptide was performed using SignalP-5.0 software (http://www.cbs.dtu.dk/services/SignalP/). The mature TaMan5 gene (without native signal peptide) was amplified from the cDNA of *T. asperellum* ND-1 with a PCR Master Mix (Thermo Scientific, USA) and the specific primers, TaMan5-F and TaMan5-R (Supplementary Table S1) using the thermal cycling conditions. The method involves initial denaturation at 95°C for 5 min, followed by 35 cycles of denaturation at 95°C for 30 s, annealing at 56°C for 30 s and extension at 72°C for 90 s, and a final extension at 72°C for 10 min. The PCR product was treated with *Xba*I and *Eco*RI and then inserted into the pPICZαA vector. The constructed plasmid was further verified by DNA sequencing analysis and named pPICZα-*TaMan5-*wt. *TaMan5* gene (without native signal peptide) was optimized according to yeast codon preference (http://www.jcat.de/), synthesized, and connected to pPICZαA vector (pPICZα-*TaMan5-*opt) by Tsingke Corp (Beijing).

The theoretical molecular weight (MW) of the mature TaMan5 was predicted by DNAMAN 6.0 software. A BLAST server was used for homology searches in the GenBank database (http://www.ncbi.nlm.nih.gov/BLAST). Multiple protein sequences were aligned using ESPript 3.0 (https://espript.ibcp.fr/ESPript/ESPript/) and Clustal Omega program (https://www.ebi.ac.uk/Tools/msa/clustalo/).

### 2.3. Transformation and fermentation in a shake flask

**Figure 1:**
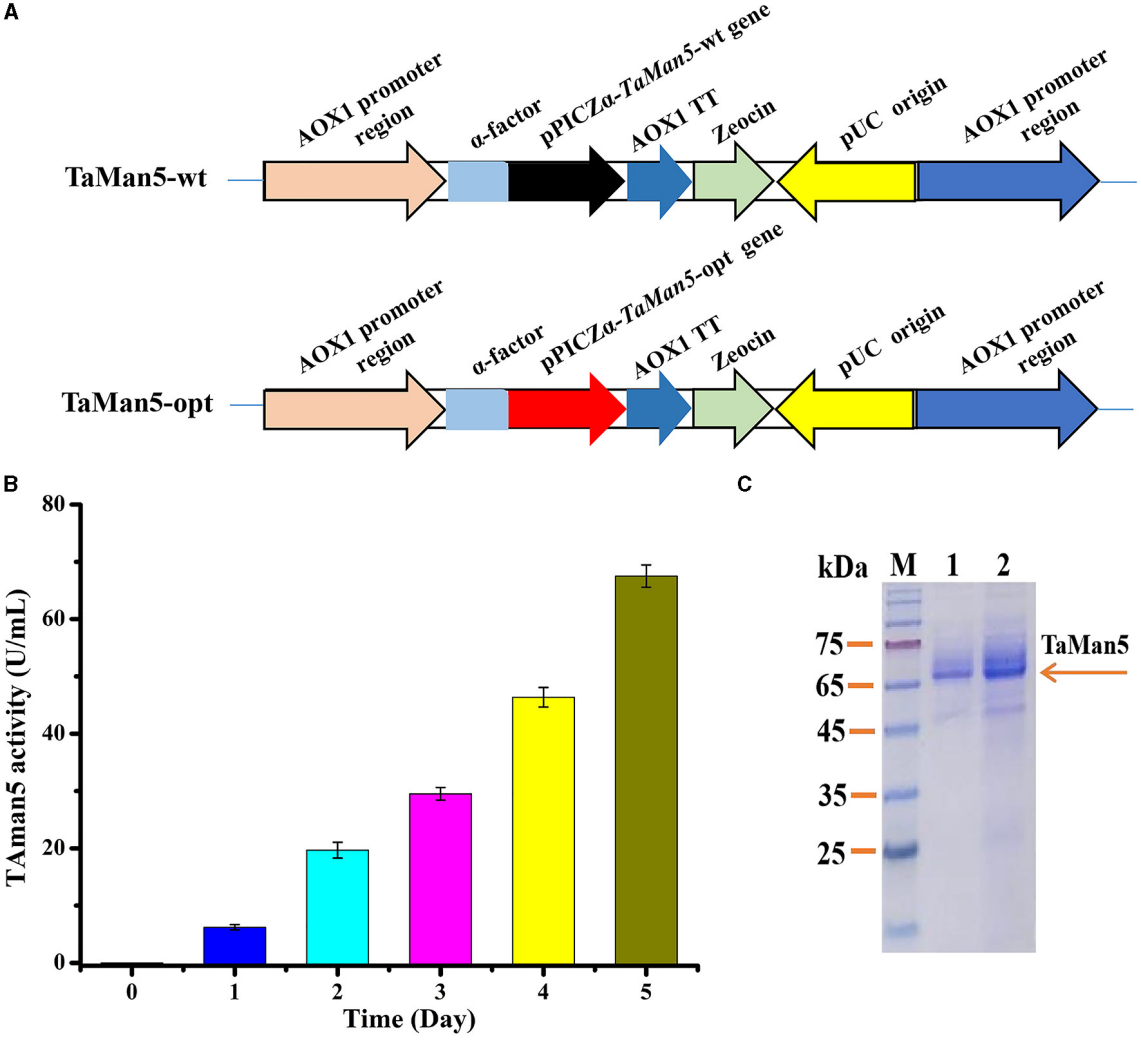
High-efficiency expression of TaMan5 in *P. pastoris*. **(A)** Schematic representation of recombinant strains TaMan5-wt and TaMan5-opt. **(B)** Extracellular enzyme activity of recombinant strain TaMan5-opt. **(C)** SDS-PAGE analysis of proteins expressed by TaMan5-wt and TaMan5-opt. Lane M: standard protein markers; Lane 1: fermentation supernatant of TaMan5-wt in shake flasks; Lane 2: fermentation supernatant of TaMan5-opt in shake flasks.

The constructed plasmids (pPICZα-*TaMan5-*wt and pPICZα-*TaMan5-*opt) were transformed into *E. coli* DH5α and extracted from transformed DH5α using a TIANprep MiNi Plasmid Kit (Tiangen, Beijing, China). pPICZαA vector contains an α-factor secretion signal for the secretion of the expressed protein. The expression vectors (pPICZα-TaMan5-wt and pPICZα-TaMan5-opt) containing α-factor signal peptide were linearized using *Sac*I, electroporated into *P. pastoris* X-33, and integrated into *Pichia*'s genome based on the homologous direct repair. The positive strains were further identified by PCR using the primer pair AOX-F/AOX-R (Supplementary Table S1) and DNA sequencing. The engineered strains were defined as TaMan5-wt and TaMan5-opt, respectively (Figure 1A). *P. pastoris* X-33 has a strong regulatory alcohol oxidase promoter (AOX1), involved in methanol utilization pathways that generate high levels of heterologous recombinant proteins (Looser et al., 2015; Taylor et al., 2018). The genotype of engineered strains was methanol utilization plus (Mut^+^).

To obtain β-mannanase TaMan5, recombinants of TaMan5-wt, TaMan5-opt, and X-33 (with an empty pPICZαA vector) were cultivated in BMMY medium with shaking flask cultivation at 28°C, 200 rpm for 5 days. During this process, the percentage content of methanol was maintained at 1.0 by adding 100% (v/v) methanol every 24 h as described previously (Basit et al., 2018). The crude culture was centrifuged (4°C, 5000 rpm) for 10 min to collect the supernatant. The expression level of TaMan5 was detected by 12% SDS-PAGE, and the protein content was analyzed using a Protein Assay Kit based on the Bradford method (Bradford, 1976). All protocols and media for *P. pastoris* were carried out by following the *Pichia* expression manual (Invitrogen, San Diego, CA).

### 2.4. Deglycosylation of recombinant TaMan5

Deglycosylation of TaMan5 was carried out with PNGase F as reported previously (Zheng et al., 2018). In brief, TaMan5 (1 mg) was mixed with denaturation buffer (pH 4.8) followed by boiling for 10 min and then treated with PNGase F (5 U) at 37°C for 2 h. The glycosylated and deglycosylated TaMan5 were detected by 12% SDS-PAGE.

### 2.5. Activity assay and biochemical characterization of TaMan5

β-Mannanase activity of TaMan5 was measured by the 3,5-dinitrosalicylic acid (DNS) method (Miller, 1959) and tested at 65°C, using LBG as the substrate. The reaction comprised 0.45 mL of sodium acetate buffer (pH 4.0) containing 0.5% (w/v) LBG and 0.05 mL of diluted enzyme. After 10 min of incubation, the reaction was terminated with 0.75 mL of DNS solution and boiled for 5 min. The absorbance of the mixture was tested at 540 nm using d-mannose as the standard. One unit (U) of β-mannanase activity was defined as the quantity of enzyme that liberated 1 μmol of reducing sugars per minute. Each experiment was performed in triplicate.

Effects of pH on TaMan5 activity were evaluated at 50°C in various 50 mM buffers including glycine–HCl buffer (pH 2.0–3.0), sodium citrate buffer (pH 3.0–4.0), sodium acetate buffer (pH 4.0–6.0), and sodium phosphate buffer (pH 6.0–8.0). The activity of TaMan5 at the optimal pH was assumed to be 100%. To estimate pH stability, TaMan5 was pre-incubated at 4°C for 1 h in the corresponding buffers (pH ranging from 2.0 to 8.0) without substrate.

The optimum temperature of TaMan5 was estimated at 20–80°C in 50 mM sodium acetate buffer (pH 4.0). Additionally, thermostability of TaMan5 was evaluated by measuring residual enzyme activity under standard assay conditions following pre-incubation at different times (1, 2, 4, 6, 8, 12, 24, and 48 h) and at 40, 50, 55, 60, 65, and 70°C without substrate. pH and temperature stability were calculated based on the ratio between residual activity and initial activity values. All experiments were conducted in triplicate independently.

The substrate specificity of TaMan5 was measured by evaluating the enzyme activity using various substrates. For polysaccharides such as LBG, GG, CMC-Na, beechwood xylan, and wheat arabinoxylan, TaMan5 activity was determined in 50 mM sodium acetate buffer (pH 4.0) containing 10 mg/mL of different substrates at 65°C by DNS method (Miller, 1959). In addition, TaMan5 activity toward 5 mM pNPAf, pNPC, and pNPG was analyzed in 50 mM sodium acetate buffer (pH 4.0) at 65 °C for 10 min and detected at 410 nm. One activity unit was defined as the amount of enzyme that released 1 μmol pNP per minute.

The kinetic parameters of TaMan5 toward LBG or GG were evaluated by testing the enzyme activities using increasing substrate concentrations ranging from 1 to 10 mg/mL. All experiments included three replicates. *V*_max_ and *K*_m_ for TaMan5 were acquired using the Lineweaver–Burk plot method (Erithacus Software, Horley, UK).

To investigate the effects of various chemical reagents and metal ions on TaMan5 activity, 1 mM, 5 mM, and 10 mM metal ions (Ni^2+^, Mg^2+^, Cd^2+^, ${\text{NH}}_{4}^{+}$, Li^+^, Fe^3+^, Al^3+^, Zn^2+^, Fe^2+^, K^+^, Ba^2+^, Ca^2+^, Pb^2+^, Cu^2+^, Co^2+^, or Mn^2+^) and chemical reagents (urea, EDTA, and SDS) were mixed with an enzyme sample in a 50-mM sodium acetate buffer (pH 4.0) at 4°C for 1 h. A sample without any additive was defined as a control (100%). The residual activity was tested using the DNS method (Miller, 1959) as described above.

### 2.6. Mode of action of TaMan5

To investigate the action model of TaMan5, a total of 5 U of TaMan5 was incubated in a reaction volume of 1 mL with 10 mg/mL of LBG, MOS (DP 2–6), and mannose at 65°C in 50 mM sodium acetate buffer (pH 4.0). Substrates containing inactive enzymes were used as blank controls. Hydrolysates (25 μL) were withdrawn at various times followed by boiling for 10 min and then detected by thin-layer chromatography (TLC) (Katrolia et al., 2013).

All samples were separated on silica gel plates (Merck Silica Gel 60F 254, Germany) with a mixture of n-propanol/ethanol/water (7:1:2, v/v/v) as a developing mobile phase. The saccharides were stained by immersing the plates with 5% sulphuric acid in methanol and subsequent heating for 15 min at 100°C. A mixture of mannose and MOS (DP 2–6) was used as the standards.

### 2.7. Construction of TaMan5 mutants

The active sites of TaMan5 were predicted to be Glu^205^ and Glu^313^ according to the BLAST results (https://blast.ncbi.nlm.nih.gov/Blast.cgi). 3D structure of TaMan5 was simulated using a SWISS-MODEL online server (https://swissmodel.expasy.org), with GH5 β-mannanase from *T. reesei* (PDB ID: 1qnr.1.A) as the template (sequence identity 57.94%) (Sabini et al., 2000). Based on the simulated 3D structure of TaMan5, residues Glu^241^, Glu^259^, Asp^152^, Asp^356^, and Asp^357^ were also considered possible catalytic sites. To further identify the crucial sites, E205A, E241A, E259A, E313A, D152A, D356A, and D357A of TaMan5 mutants were obtained by site-directed mutagenesis corresponding to Glu^205^, Glu^241^, Glu^259^, Glu^313^, Asp^152^, Asp^356^, and Asp^357^ residues. Constructed plasmids possessing the mutant genes were amplified from the expression vectors pPICZα-*TaMan5-*opt with the specific primers (Supplementary Table S1) according to the previous report (Basit et al., 2019). Protein expression and activity tests were performed as described above. *P. pastoris* possessing the normal gene (*TaMan5*-opt) was defined as the blank control.

### 2.8. Synergistic action of TaMan5 and commercial α-galactosidase on the degradation of galactomannans

To evaluate the synergistic action of TaMan5 and commercial α-galactosidase (termed AnAgal) on the degradation of galactomannans, 10 mg/mL of LBG or GG degraded by TaMan5 (15 U/mL) and AnAgal (25 U/mL) was performed sequentially or simultaneously. The degree of synergy was calculated by testing the amounts of reducing sugars produced by the hydrolysis action. The reactions (5 mL) were incubated in 50 mM sodium acetate buffer (pH 4.0) at 60 °C, mixing at 200 rpm for 6 h in simultaneous actions, and then stopped by boiling for 5 min. For the sequential action, the first enzyme was interrupted by boiling for 5 min after hydrolysis for 6 h. Then, another enzyme was loaded, and the reaction was stopped by boiling for 5 min after hydrolysis for 6 h under the same conditions. Reactions containing substrate alone were used as blank controls. Supernatants were collected by centrifuging at 12000 rpm for 3 min. Amounts of reducing sugars in the supernatant were tested using the DNS method with mannose as the standard (Miller, 1959).

## 3. Results and discussion

### 3.1. Sequence analysis of *TaMan5* gene

The β-mannanase gene (*TaMan5*) from *T. asperellum* ND-1 encodes 446 amino acids with an open reading frame (ORF) comprising 1341 bp (Gene ID: OM128445). After codon optimization, the codon adaptation index was enhanced from 0.66 to 0.94, and the GC content of the *TaMan5* gene was reduced from 50.2 to 42.0% (Supplementary Figure S1). Three potential N-glycosylation sites (Asn-Xaa-Ser/Thr) of TaMan5 were found using the NetNGlyc 1.0 (http://www.cbs.dtu.dk/services/NetNGlyc/). The SignalP-5.0 software analysis suggested that TaMan5 harbored a predicted signal peptide of 27 residues (MGNRALNSMKFFKSQALALLAATSAVA).

**Figure 2:**
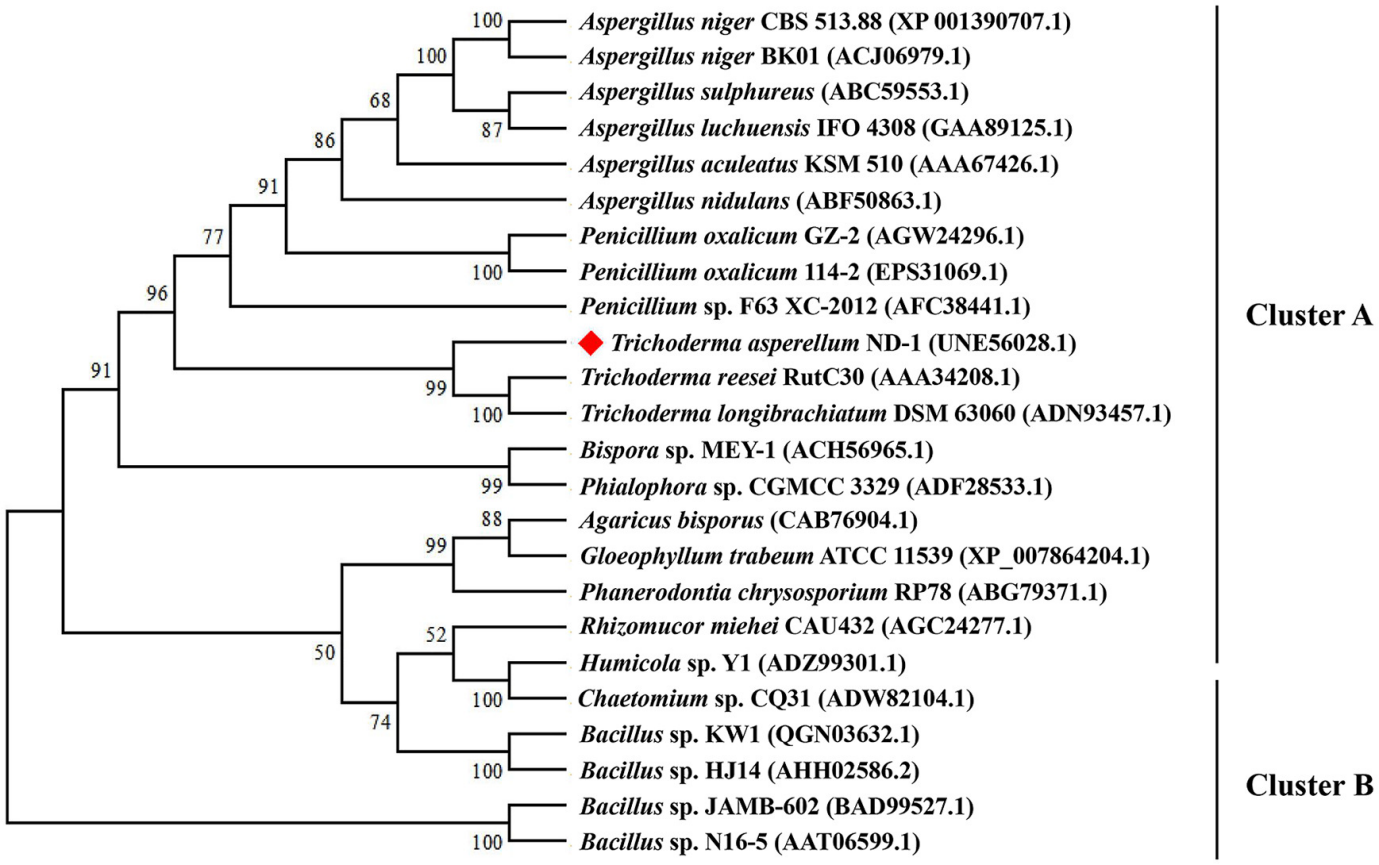
Phylogenetic relationships of the amino acid sequences of TaMan5 from *T. asperellum* ND-1 and the reference sequences of GH5 β-mannanases in GenBank. The phylogenetic tree was built using the neighbor-joining method. Numbers at the node are the bootstrap values (%).

Phylogenetic analysis (Figure 2) showed that TaMan5 and the other 23 β-mannanases belonging to the GH5 family were divided into two groups marked as A and B, respectively. Among them, group B consisted of 5 sequences from bacteria, but group A comprised 19 sequences from fungi including TaMan5. Additionally, the sequence alignment showed that TaMan5 displayed maximum similarity with the GH5 family β-mannanase from *T. reesei* RutC30 (69.57%, AAA34208.1), followed by the GH5 family β-mannanase from *T. reesei* (58.17%, PDB ID code: 1qnr.1.A) (Sabini et al., 2000), *A. niger* BK01 (47.65, ACJ06979.1) (Do et al., 2009), and *Bacillus* sp. KW1 (27.63%, QGN03632.1) (Chen et al., 2021) (Supplementary Figure S2).

Therefore, TaMan5 should be a novel GH5 family β-mannanase on the basis of the phylogenetic analysis and multiple protein sequences alignment, suggesting its maximum closeness with the β-mannanases from *Trichoderma* species (Figure 2). Meanwhile, two catalytic sites (E205 and E313) of TaMan5 were predicted online (https://www.ncbi.nlm.nih.gov/Structure/cdd/wrpsb.cgi), which were highly conserved in the other GH5 β-mannanases (Supplementary Figure S2).

### 3.2. Heterologous expression and deglycosylation of TaMan5

Various strategies have been developed to satisfy the high-level production of target proteins in *P. pastoris* (Yang and Zhang, 2018). Particularly, codon optimization of the target gene expressed in *P. pastoris* is an effective and preferable way (Looser et al., 2015). Hence, the *TaMan5* gene from *T. asperellum* ND-1 was optimized according to the preferred codon usage in yeast. The codon-optimized gene (*TaMan5-*opt) showed 76.44% similarity with the native gene (*TaMan5-*wt), with 316 nucleotides being modified (Supplementary Figure S1). The positive strains, TaMan5*-*wt and TaMan5*-*opt (Figure 1A), were verified using PCR (Supplementary Figure S3). Wild-type strain X-33 with an empty pPICZαA vector was used as blank control.

After 5 days of induction by 1% (v/v) methanol, the TaMan5 activity of TaMan5*-*opt fermentation supernatant continuously improved, reaching a maximum of 67.5 ± 1.95 U/mL (Figure 1B), which was about twice that of TaMan5*-*wt (35.75 ± 0.86 U/mL). Moreover, the specific activity of TaMan5 produced by constructed strain TaMan5*-*opt (718.14 ± 20.76 U/mg) toward LBG was remarkably higher than β-mannanases from *Bacillus* sp. HJ14 (443.4 ± 8.5 U/mg) (Zhang et al., 2016), *Ruminococcus flavefaciens* (298.5 U/mg) (Goyal et al., 2019), and *Penicillium oxalicum* GZ-2 (420.9 U/mg) (Liao et al., 2014). It has been reported that heterologous proteins can efficiently improve the expression levels in *P. pastoris* via codon optimization, such as the α-galactosidase of *Lichtheimia ramosa* (Xie et al., 2020), cellobiohydrolase of *Lentinula edodes* (Li et al., 2019), and α-l-arabinofuranosidase of *A. niger* ND-1 (Zheng et al., 2018).

**Figure 3:**
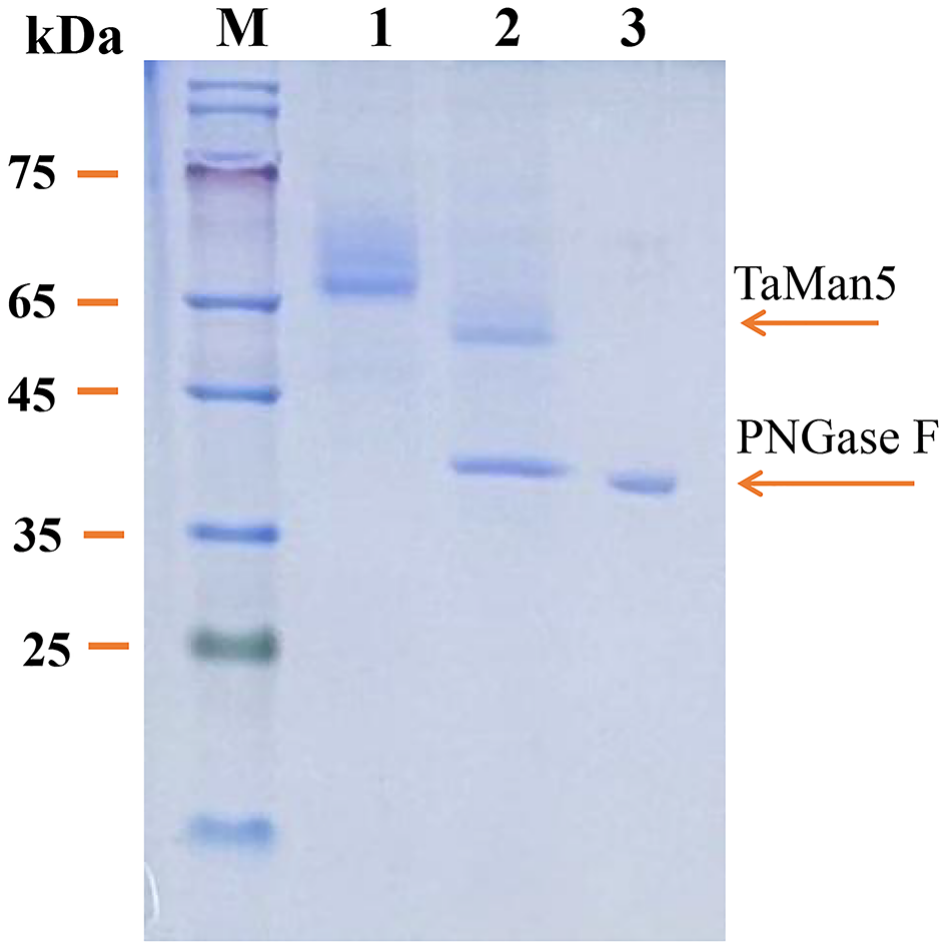
SDS-PAGE analysis of recombinant TaMan5 produced by TaMan5-opt following deglycosylation. M, marker; 1, glycosylated TaMan5; 2, deglycosylated TaMan5; 3, PNGase F.

The SDS-PAGE analysis revealed that the culture supernatant of TaMan5*-*opt and TaMan5*-*wt migrated as a single band with an approximate MW of 66.0 kDa (Figure 1C). It has been reported that MW values of β-mannanases from different sources are in the range of 30–80 kDa (Srivastava and Kapoor, 2017). Interestingly, the MW of TaMan5 determined by SDS-PAGE was much bigger than its theoretical value (47.4 kDa), suggesting that TaMan5 is a glycoprotein. Glycosylation is a crucial form of protein post-translational modification that could affect the structure and activity of enzymes expressed in *P. pastoris* (Bretthauer, 2003; Amore et al., 2017). Three N-glycosylation sites (Asn^149^, Asn^166^, and Asn^268^) of TaMan5 were found using the NetNGlyc 1.0 (http://www.cbs.dtu.dk/services/NetNGlyc/). More specifically, Asn^149^, Asn^166^, and Asn^268^ were located on the surface of the TaMan5 protein, and Asn^149^ was close to the active site Glu^205^ (Supplementary Figure S4). N-glycosylation is obligatory for viability. It functions by modifying appropriate asparagine residues of proteins with oligosaccharide structures, thus influencing their properties and bioactivities (Ge et al., 2018; Karbalaei et al., 2020). Many studies have proven that N-glycosylation plays an important role in the activity, thermostability, and secretion of recombinant enzymes in yeast (Hoshida and Fujita, 2013; Yang et al., 2015). Han et al. introduced N-glycosylation at sites 36, 67, and 264 in elastase expressed by *P.pastoris* and found that the A69T mutation completely inhibited the secretion of recombinant elastase and that the I38T mutant had increased specific activity and similar activity but slightly improved thermostability were observed in the N266T mutant (Han et al., 2014). After deglycosylation with PNGase F, the MW of TaMan5 was close to the theoretical value (Figure 3), and its activity was decreased by 55.37%, which was similar to the reported findings for other fungal hemicellulases, *e.g.*, β-xylanase, α-galactosidase, and α-l-arabinofuranosidase (Miller, 1959; Birijlall et al., 2011).

### 3.3. Effect of pH and temperature on the activity of TaMan5

**Figure 4:**
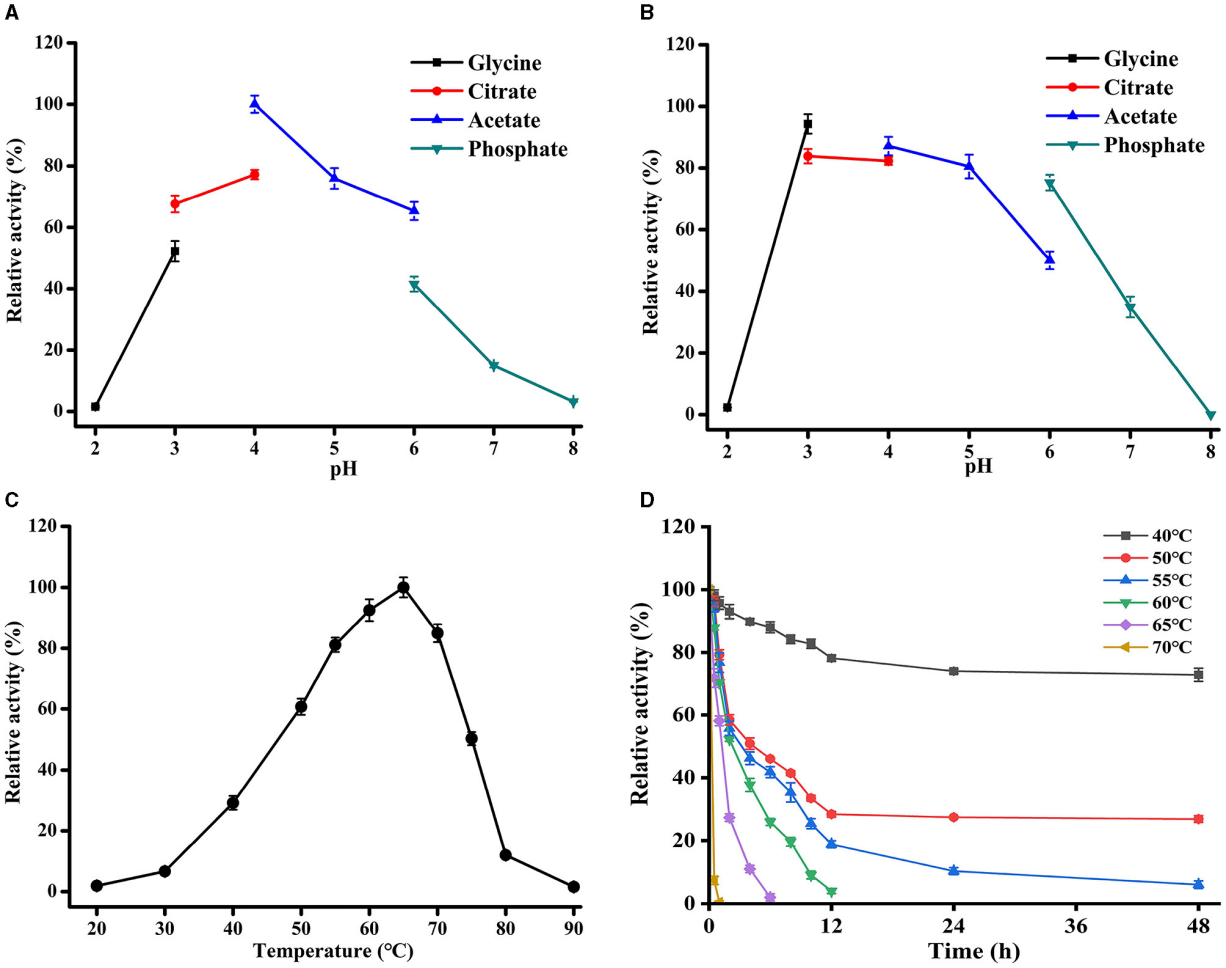
Effects of pH and temperature on TaMan5 activity. **(A)** Optimal pH; **(B)** pH stability; **(C)** optimal temperature; and **(D)** temperature stability. All experiments were carried out in triplicate.

The effects of pH and temperature on the activity of TaMan5 expressed by TaMan5*-*opt were investigated using 5 mg/mL of LBG. TaMan5 exhibited the highest activity at pH 4.0 in sodium acetate buffer; over 65% of the maximum activity was maintained in pH 3.0–5.0, suggesting that the enzyme is an acidophilic β-mannanase (Figure 4A). In general, most of the fungal GH5 β-mannanases exhibit pH optima in an acidic range, such as β-mannanases from *T. reesei* (pH 3.5) (Eneyskaya et al., 2009), *A. aculeatus* (pH 2.5) (Pham et al., 2010), and *P. oxalicum* GZ-2 (pH 4.0) (Liao et al., 2014). Furthermore, TaMan5 exhibited remarkable tolerance to an acidic environment, retaining over 80% of its original activity at pH 3.0–5.0 after pretreatment for 1 h at 4°C (Figure 4B).

The optimal temperature of TaMan5 was 65°C, with more than 80% of its peak activity observed between 55 and 70°C (Figure 4C). Nevertheless, the enzymatic activity rapidly decreased when the temperature was above 65°C, and the relative activities were <20% at temperatures ≥ 80°C (Figure 4C). With respect to thermostability, TaMan5 was excellently stable for 30 min at 40–60°C, maintaining over 80% of its initial activity (Figure 4D). However, the enzyme activity reduced rapidly and was completely inactivated after treatment for 30 min at above 65^o^C (Figure 4D). Taking into account all of the above, TaMan5 could be a great candidate for MOS preparation and application in the feed industry due to its excellent acidic-tolerant and thermostability.

### 3.4. Substrate specificity and kinetic parameters of TaMan5

Substrate specificities of TaMan5 were determined using various substrates, including LBG, GG, beechwood xylan, CMC-Na, pNPC, pNPAf, and pNPG. The results are presented in Table 1. TaMan5 exhibited strict substrate specificity toward galactomannan. It displayed the maximum activity for LBG (67.5 ± 1.95 U/mL, 100%), followed by GG (41.32 ± 1.25 U/mL, 61.2%), which was similar to other GH5 β-mannanases from *Bacillus* sp. KW1 (Chen et al., 2021), *P. oxalicum* GZ-2 (Liao et al., 2014), and *R. miehei* (Katrolia et al., 2013). Moreover, TaMan5 showed minor activity (3.4 ± 0.25 U/mL, 5.1%) toward CMC-Na, but no detectable activity for the hydrolysis of beechwood xylan, wheat arabinoxylan, and pNP derivatives (pNPC, pNPAf, and pNPG).

**Table 1:** Hydrolysis of different substrates catalyzed by TaMan5.

**Substrate**	**Concentration [1% (w/v)]**	**Relative activity^a^ (%) (mean ±SD, N = 3)**
Locust bean gum (LBG)	0.5	100 ± 1.95
Guar gum (GG)	0.5	61.2 ± 0.64
Sodium carboxymethyl cellulose (CMC-Na)	1.0	5.1 ± 0.13
Beechwood xylan	1.0	1.6 ± 0.03
Wheat arabinoxylan	1.0	0.5 ± 0.02
p-Nitrophenyl-α-L-arabinofuranoside (pNPAf)	5 mM	0.0 ± 0.00
p-Nitrophenyl-β-D-cellobioside (pNPC)	5 mM	0.0 ± 0.00
p-Nitrophenyl-β-D-galactopyranoside (pNPG)	5 mM	0.0 ± 0.00

^a^Relative activities were calculated in relation to LBG activity, which was considered 100%.

The kinetic parameters of the TaMan5 were evaluated at 65°C and pH4.0 using LBG and GG as the substrates. *K*_m_ and *V*_max_ values were 1.34 mg/mL and 749.14 μmol/min/mg for LBG, and 3.21 mg/mL and 578.16 μmol/min/mg for GG, respectively. Moreover, the *K*_m_ value of TaMan5 revealed a greater affinity for LBG in comparison to the *K*_m_ reported for the other GH5 fungal β-mannanases, such as rManAJB13 from *Sphingomonas* sp. JB13 (5.0 mg/mL) (Zhou et al., 2012), RmMan5A from *R. miehei* CAU432 (3.78 mg/mL) (Katrolia et al., 2013), and ManAC from *A. calidoustus* (3.4 mg/mL) (Sun et al., 2021).

### 3.5. Effect of metal ions and chemical reagents on the activity of TaMan5

The effects of different metal ions and chemicals on TaMan5 activity are shown in Table 2. The presence of metal ions at 1 or 5 mM (Ni^2+^, Zn^2+^, Al^3+^, Co^2+^, and Ba^2+^) had partial or no effect on TaMan5 activity (Table 2). Li^+^ (1 mM, 108.60%), Fe^2+^ (1 mM, 114.96%), K^+^ (1 mM, 109.18%), and Ca^2+^ (1 mM, 118.05%) slightly or significantly promoted the activity of TaMan5 (Table 2). Upon increasing the concentration to 5 mM, TaMan5 activity was further improved by Fe^2+^ (118.55%) and Fe^3+^ (113.98%), respectively (Table 2). The residue metal ions investigated in this study showed a negative effect on TaMan5 activity (Table 2). Among them, Mn^2+^ and Cu^2+^ significantly decreased TaMan5 activity by 55.91% and 61.94%, respectively (Table 2). Moreover, TaMan5 activity was significantly decreased by 10 mM Mg^2+^ (56.36%), Mn^2+^ (51.31%), Ca^2+^ (73.61%), and Fe^3+^ (29.19%), respectively. Ni^+^, Li^+^, Fe^2+^, and Cd^2+^ remarkably increased TaMan5 activity by 45.2%, 26.73%, 68.02%, and 92.76%, respectively (Table 2). Additionally, TaMan5 activity was intensely inhibited, and no activity was detected in the presence of 1, 5, or 10 mM SDS, as reported previously for β-mannanases from *A. niger* BK01 (20.5%) (Do et al., 2009), *Sphingomonas* sp. JB13 (0%) (Zhou et al., 2012) and *B. subtilis* WY34 (55.5%) (Jiang et al., 2006). On the contrary, 1, 5, or 10 mM EDTA and urea have little effect on TaMan5 activity, suggesting that TaMan5 is not a metaloenzyme and could tolerate urea.

**Table 2:** Effects of metal ions and chemical reagents on TaMan5 activity.

**Chemicals**	**Relative activity (%)**		
	**1 mM**	**5 mM**	**10 mM**
Control	100.00 ± 0.21	100.00 ± 0.49	100.00 ± 0.156
Mg^2+^	94.14 ± 1.43	64.40 ± 0.42	43.64 ± 1.54
NH^4+^	101.66 ± 0.42	79.38 ± 1.30	103.74 ± 0.85
Ni^2+^	95.68 ± 0.67	91.82 ± 0.79	145.20 ± 0.33
Li^+^	108.60 ± 1.59	97.85 ± 0.44	126.73 ± 4.82
Fe^2+^	114.96 ± 1.85	118.55 ± 0.24	168.02 ± 1.95
K^+^	109.18 ± 0.53	77.53 ± 0.50	101.30 ± 0.78
Cd^2+^	97.03 ± 2.14	65.09 ± 0.44	192.76 ± 6.60
Zn^2+^	85.46 ± 0.42	93.86 ± 1.24	92.93 ± 0.76
Al^3+^	79.39 ± 1.20	80.83 ± 0.76	72.21 ± 0.84
Mn^2+^	85.56 ± 0.13	44.09 ± 0.87	48.69 ± 0.48
Pb^2+^	104.26 ± 0.14	62.85 ± 0.53	82.48 ± 0.64
Ca^2+^	118.05 ± 0.63	104.26 ± 0.21	26.39 ± 1.26
Co^2+^	88.74 ± 2.32	77.33 ± 1.7	91.37 ± 0.86
Ba^2+^	103.40 ± 1.13	103.69 ± 2.36	95.90 ± 0.70
Cu^2+^	82.96 ± 0.91	38.06 ± 1.84	82.83 ± 0.5
Fe^3+^	101.18 ± 1.71	113.98 ± 3.25	70.81 ± 0.55
Urea	88.64 ± 1.38	79.45 ± 2.98	91.37 ± 1.22
SDS	0.00	0.00	0.00
EDTA	88.35 ± 1.86	95.42 ± 1.26	89.98 ± 2.03

### 3.6. Mode of action of TaMan5

**Figure 5:**
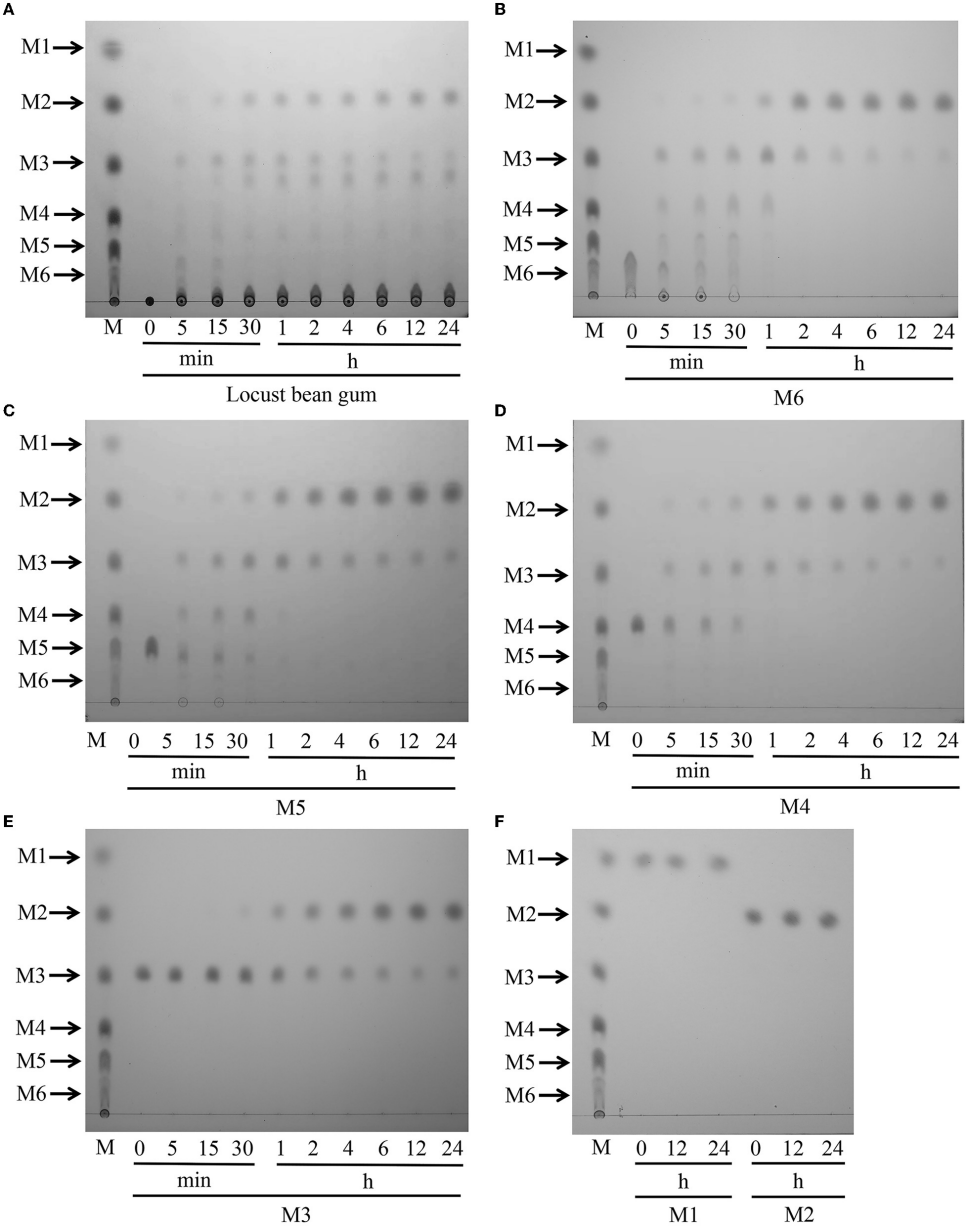
Hydrolytic activity of TaMan5 by TLC analysis. **(A)** Time-course hydrolysis of LBG by TaMan5. **(B–F)** TLC analysis of hydrolysis products of MOS (DP 2–6) and mannose catalyzed by TaMan5. Mannohexaose (M6), mannopentaose (M5), mannotetraose (M4), mannotriose (M3), mannobiose (M2), and mannose (M1).

The products obtained by hydrolyzing LBG with 5 U of TaMan5 were investigated by TLC. As shown in Figure 5, during the continuous degradation of LBG (Figure 5A), the length of yielded oligosaccharides was shorter, and the end-products were mainly mannobiose and mannotriose. Similarly, other β-mannanases from fungi such as *A. niger* BK01 (Do et al., 2009), *R. miehei* CAU432 (Katrolia et al., 2013), and *P. chrysosporium* (Benech et al., 2007) hydrolyze mannan to generate mainly mannobiose. Mannobiose can be used directly as a food additive and selectively promote the growth of beneficiary bacteria, such as *Bifidobacteria* sp. and *Lactobacillus* sp. (Hlalukana et al., 2021).

To study the action mode of TaMan5, the hydrolysis products of mannose and MOS (DP 2–6) were assessed. The results displayed that TaMan5 could rapidly degrade mannohexaose, mannopentaose, mannotetraose, and mannotriose, yielding mainly mannobiose (Figures 5B–E). It is worth noting that, although M2 was found during the degradation of M3, the formation of a correspondent amount of M1 was not detected (Figure 5E), indicating that TaMan5 possessed transglycosylation capacity. Several GH5 β-mannanases have also been reported to perform the transglycosylation reactions, e.g., PaMan5A from *Podospora anserina* (Couturier et al., 2013), rManH from *Cellulosimicrobium* sp. HY-13 (Kim et al., 2011), and AnMan5B from *A. nidulans* (Rosengren et al., 2014). Moreover, TaMan5 exhibited no detectable activity toward mannobiose (Figure 5F), suggesting that it was a typical endo-acting β-mannanase. It is well known that enzymes with much lower β-mannosidases or exo-mannanases activity are attractive tools (Gómez et al., 2017; Jana et al., 2021); hence, TaMan5 is more suitable for the preparation of mannobiose-enriched MOS prebiotics, which can promote the growth of human-beneficial intestinal microflora.

### 3.7. Mutant construction for the catalytic sites of TaMan5

**Figure 6:**
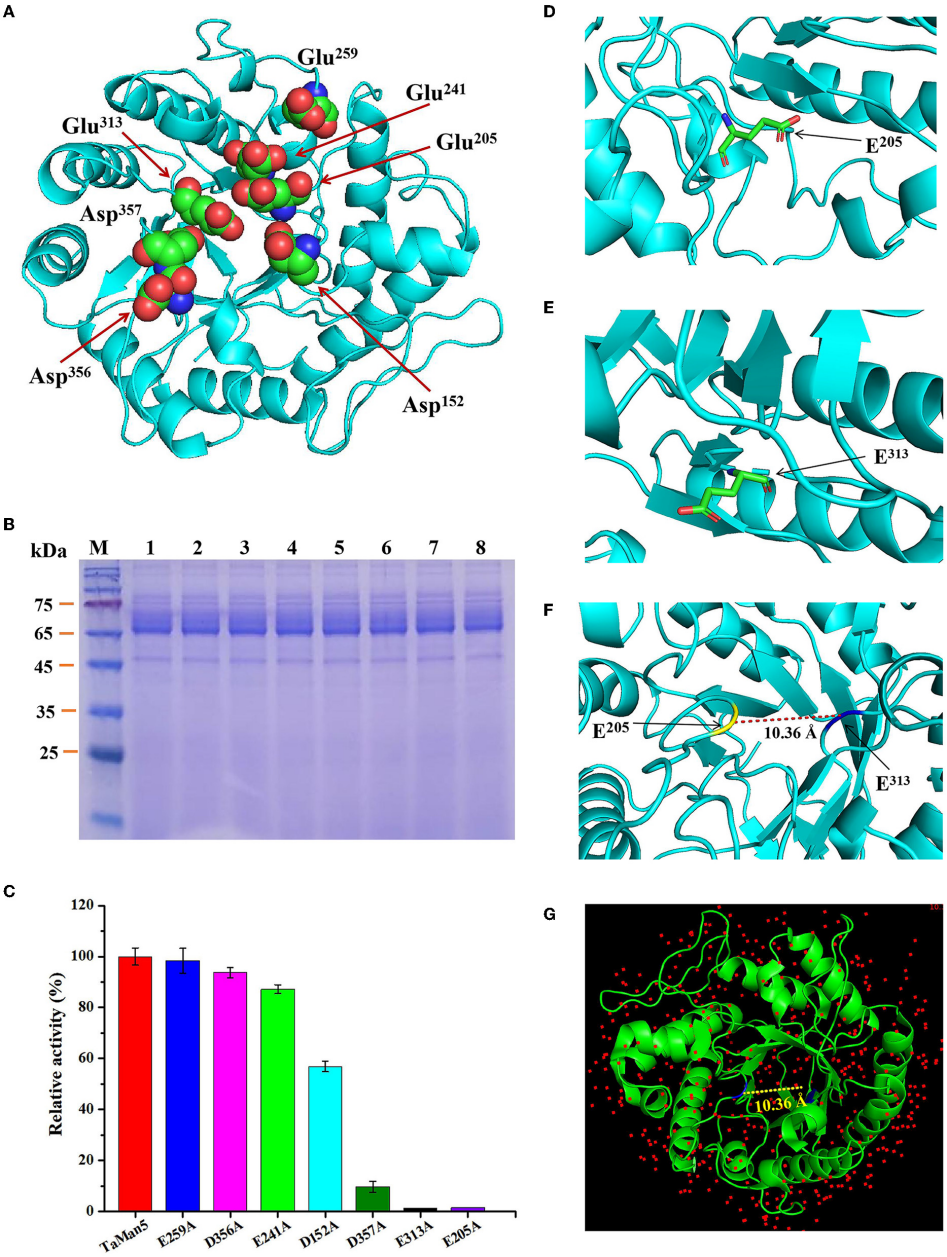
Confirmation of TaMan5 catalytic sites. **(A)** 3D structure of enzyme simulated by SWISS-Model software. Predicted catalytic sites are marked. SDS-PAGE analysis **(B)** and enzyme activities **(C)** of TaMan5 and its mutants. The amino acid residue E205 **(D)** and E313 **(E)** was labeled. **(F)** The two residues in TaMan5 protein are separated by approximately 10.36 Å. **(G)** The two residues in the structure of template (1qnr.1.A) are located at 10.36 Å.

According to TaMan5, a three-dimensional structure was simulated by using *T. reesei* β-mannanase (PDB ID: 1qnr.1.A) as the template (Sabini et al., 2000), and potential catalytic sites of TaMan5 were selected (Figure 6A) and mutated by site-directed mutagenesis to confirm vital residues responsible for enzyme activity. The SDS-PAGE analysis displayed that TaMan5 and its mutants migrated as a single band at approximately 66.0 kDa (Figure 6B). The enzyme activities of mutants E241A, E259A, and D356A were very close to normal β-mannanase TaMan5 (Figure 6C, Table 3). However, mutants of D152A and D357A led to a drastic decrease (by ~40 and 90%, respectively) in TaMan5 activity (Figure 6C, Table 3), indicating the auxiliary function of Asp^152^ and Asp^357^ toward mannan deconstruction. Additionally, E205A and E313A were located at the catalytic domain of TaMan5 (Figures 6D, E), and their distance was approximately 10.36 Å (Figure 6F), which is similar to the distance between the conserved sites in the structure of 1qnr.1.A (10.36 Å) (Figure 6G). Furthermore, no enzyme activity was found for the mutants E313A and E205A (Figure 6C), indicating that Glu^313^ and Glu^205^ were the critical sites for TaMan5 activity. Chen et al. reported that Glu^206^ and Glu^314^ were the necessary catalytic residues of β-mannanase *A. sulphureus* and they also did not exhibit any activity (Chen et al., 2008).

**Table 3:** Enzyme activities of TaMan5 and its mutants.

**TaMan5 mutants**	**Relative activity (%)**
Control	100 ± 3.41
E259A	98.36 ± 4.87
D356A	93.72 ± 2.08
E241A	87.19 ± 1.75
D152A	56.82 ± 2.03
D357A	9.61 ± 2.16
E313A	1.14 ± 0.04
E205A	1.45 ± 0.03

### 3.8. Degradation of galactomannans by the synergistic action of TaMan5 and commercial α-galactosidase

High-efficiency hydrolysis of galactomannans demands that multiple enzymes, particularly β-mannanases and α-galactosidases (Moreira and Filho, 2008; Wang et al., 2010; Malgas et al., 2015), cooperatively act together. Moreover, the complete conversion of galactomannan main chains to mannobiose depends on various factors, such as the ratio of mannose to galactose, and the simultaneous or sequential action of enzymes. As the final hydrolysate of mannan, mannobiose can be used as a prebiotic and promote the growth of beneficial bacteria such as *Lactobacillus* sp. and *Bifidobacteria* sp. (Hlalukana et al., 2021).

**Figure 7:**
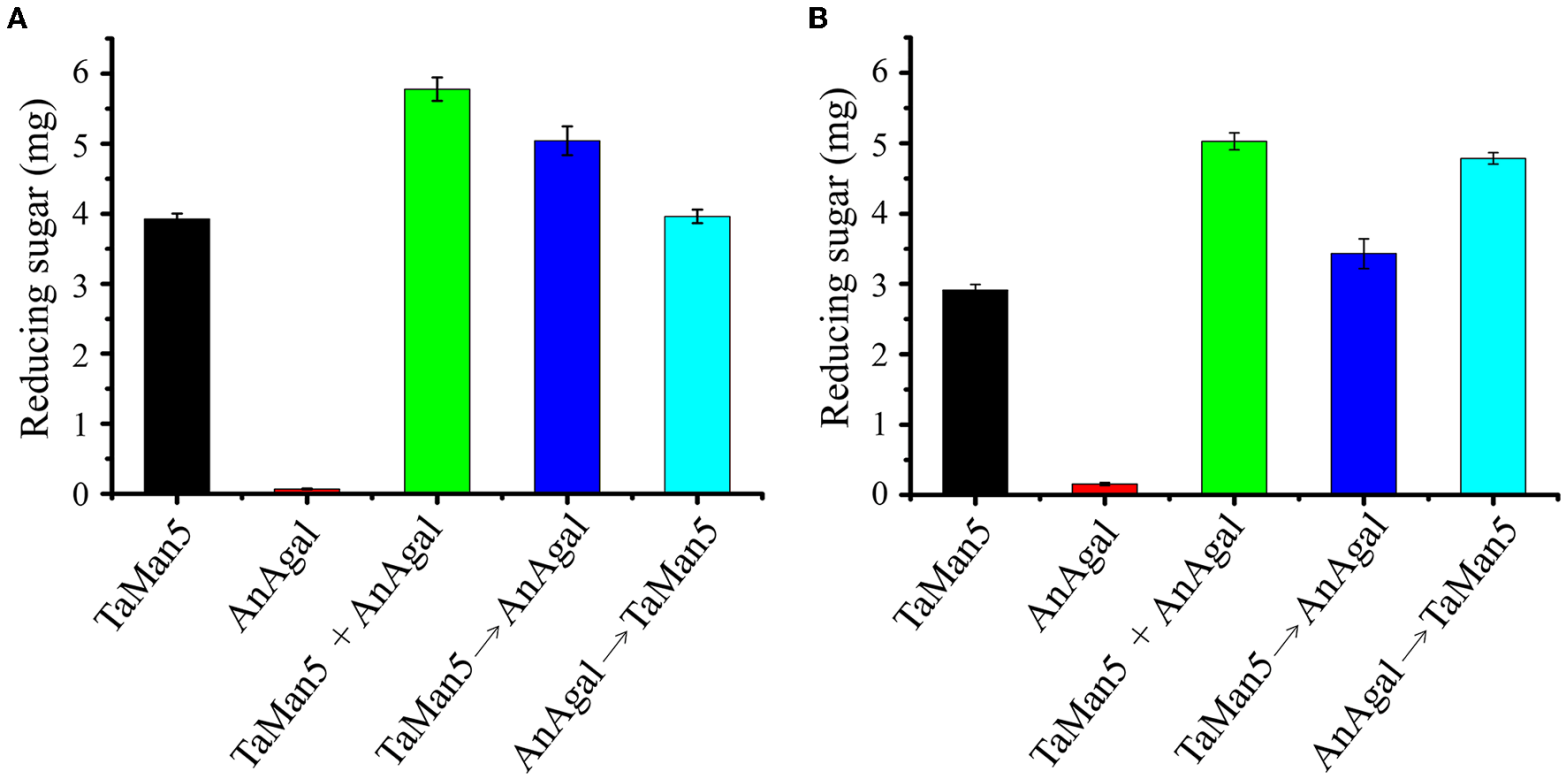
Synergistic action of TaMan5 and commercial α-galactosidase AnAgal on the degradation of galactomannans. **(A)** LBG; **(B)** GG. Data reflect the mean ± SD (n = 3).

The capability of TaMan5 and commercial α-galactosidase AnAgal individually or in combination with hydrolyzed LBG or GG was evaluated under simulated gastric conditions (sodium acetate buffer, pH 4.0). As shown in Figure 7, simultaneous reactions (TaMan5 + AnAgal) exhibited a remarkable synergistic effect on the hydrolysis of LBG or GG, compared to the sum of reducing sugar released by TaMan5 or AnAgal alone. The release of reducing sugar increased by 44.9% and 66.7%, respectively (Figure 7). In addition, the effects of the sequential addition of TaMan5 and AnAgal on LBG or GG degradation were further investigated. When LBG was initially degraded by TaMan5 for 6 h and then treated with AnAgal for another 6 h, the reducing sugar yield was close to that following the simultaneous action of the enzymes (Figure 7A). This phenomenon is similar to α-galactosidase and β-mannanase from *L. ramosa* (Xie et al., 2020). Moreover, the sequential degradation (AnAgal → TaMan5) for GG improved the release of reducing sugar by 59.3% (Figure 7B), which was much higher than that of LBG (Figure 7A). The reason for this may be that galactomannans contain different ratios of mannose to galactose (M:G); and LBG has a higher M:G ratio (4:1) than that of GG (2:1) (Malgas et al., 2015; Singh et al., 2018). In the degradation process, AnAgal catalyzes the cleavage α-1,6-linked galactose residues from the side chain of GG, which can remarkably promote accessibility of the mannan backbone to TaMan5, thereby exhibiting much higher hydrolysis efficiency (Moreira and Filho, 2008; Aulitto et al., 2019; Chen et al., 2021). The synergism of TaMan5 and commercial α-galactosidases could efficiently decrease costs and energy required in terms of improving prebiotic mannobiose production, which was an environmentally friendly and sustainable strategy to produce value-added bioactive polysaccharide from agricultural waste.

## 4. Conclusion

A novel β-mannanase (TaMan5) from *T. asperellum* ND-1 displayed multiple properties of acidophilic, ethanol-tolerant and halophilic was characterized and effectively improved its activity via codon optimization. TaMan5 can rapidly convert mannan into mannobiose without mannose via hydrolysis activity and transglycosylation activity. Site-directed mutagenesis results showed that Glu^205^, Glu^313^, and Asp^357^ of TaMan5 are the crucial catalytic residues, with Asp^152^ playing an auxiliary function. In addition, the synergy of TaMan5 and commercial α-galactosidases could remarkably enhance the yield of bioactive polysaccharide mannobiose. This study provided a novel acidophilic β-mannanase with ideal characteristics and can, therefore, be considered a potential candidate for application in food and animal feed industries.

## Data availability statement

The datasets presented in this study can be found in online repositories. The names of the repository/repositories and accession number(s) can be found in the article/Supplementary material.

## Author contributions

FZ conceived and designed the research, supervised the project, and revised the manuscript. FZ and AB analyzed the data and wrote the manuscript. FZ, JW, HZ, JC, and JZ performed the experiments and analyzed the data. All authors contributed to the article and approved the submitted version.

## References

[B1] Amore A. Knott B. C. Supekar N. T. Shajahan A. Azadi P. Zhao P.. (2017). Distinct roles of N- and O-glycans in cellulase activity and stability. Proc. Natl. Acad. Sci. USA. 114, 13667–13672. 10.1073/pnas.1714249114 29229855 PMC5748201

[B2] Arnling Bååth J. Martínez-Abad A. Berglund J. Larsbrink J. Vilaplana F. Olsson L.. (2018). Mannanase hydrolysis of spruce galactoglucomannan focusing on the influence of acetylation on enzymatic mannan degradation. Biotechnol. Biofuels. 11, 114. 10.1186/s13068-018-1115-y 29713374 PMC5907293

[B3] Aulitto M. Fusco S. Limauro D. Fiorentino G. Bartolucci S. Contursi P.. (2019). Galactomannan degradation by thermophilic enzymes: a hot topic for biotechnological applications. World. J. Microbiol. Biotechnol. 35, 32. 10.1007/s11274-019-2591-3 30701316

[B4] Barak S. Mudgil D. (2014). Locust bean gum: processing, properties and food applications–a review. Int. J. Biol. Macromol. 66, 74–80. 10.1016/j.ijbiomac.2014.02.017 24548746

[B5] Basit A. Liu J. Miao T. Zheng F. Rahim K. Lou H.. (2018). Characterization of two endo-β-1,4-xylanases from *Myceliophthora. thermophila* and their saccharifification efficiencies, synergistic with commercial cellulase. Front. Microbiol. 9, 233. 10.3389/fmicb.2018.00233 29491860 PMC5817056

[B6] Basit A. Miao T. Liu J. Wen J. Song L. Zheng F.. (2019). Highly efficient degradation of xylan into xylose by a single enzyme. ACS. Sustainable. Chem. Eng. 7, 11360–11368. 10.1021/acssuschemeng.9b00929

[B7] Behera S. Dev M. J. Singhal R. S. (2022). Cross-linked β-mannanase aggregates: preparation, characterization, and application for producing partially hydrolyzed guar gum. Appl. Biochem. Biotechnol. 194, 1981–2004. 10.1007/s12010-022-03807-w 35006550

[B8] Benech R. O. Li X. Patton D. Powlowski J. Storms R. Bourbonnais R.. (2007). Recombinant expression, characterization, and pulp prebleaching property of a *Phanerochaete chrysosporium* endo-β-1,4-mannanase. Enzyme. Microb. Technol. 41, 740–747. 10.1016/j.enzmictec.2007.06.012

[B9] Birijlall N. Manimaran A. Kumar K. S. Permaul K. Singh S. (2011). High level expression of a recombinant xylanase by *Pichia. pastoris* NC38 in a 5 L fermenter and its efficiency in biobleaching of bagasse pulp. Bioresour. Technol. 102, 9723–9729. 10.1016/j.biortech.2011.07.059 21852117

[B10] Bradford M. M. A. (1976). rapid and sensitive method for the quantitation of microgram quantities of protein utilizing the principle of protein-dye binding. Anal. Biochem. 72, 248–254. 10.1016/0003-2697(76)90527-3 942051

[B11] Bretthauer R. K. (2003). Genetic engineering of *Pichia. pastoris* to humanize N-glycosylation of proteins. Trends. Biotechnol. 21, 459–462. 10.1016/j.tibtech.2003.09.005 14573354

[B12] Chauhan P. S. Puri N. Sharma P. Gupta N. (2012). Mannanases: microbial sources, production, properties and potential biotechnological applications. Appl. Microbiol. Biotechnol. 93, 1817–1830. 10.1007/s00253-012-3887-5 22314515

[B13] Chen X. Lu W. Cao Y. Prokaryotic L. i. D. (2008). expression, purification and characterization of *Aspergillus sulphureus* β-mannanase and site-directed mutagenesis of the catalytic residues. Appl. Biochem. Biotechnol. 149, 139–144. 10.1007/s12010-007-8037-7 18401744

[B14] Chen X. Wang X. Liu Y. Zhang R. Zhang L. Zhan R.. (2021). Biochemical analyses of a novel thermostable GH5 endo β-1,4-mannanase with minor β-1,4-glucosidic cleavage activity from *Bacillus* sp. KW1 and its synergism with a commercial α-galactosidase on galactomannan hydrolysis. Int. J. Biol. Macromol. 166, 778–788. 10.1016/j.ijbiomac.2020.10.235 33144255

[B15] Couturier M. Roussel A. Rosengren A. Leone P. Stålbrand H. Berrin J. G.. (2013). Structural and biochemical analyses of glycoside hydrolase families 5 and 26 β-(1,4)-mannanases from *Podospora. anserina* reveal differences upon manno-oligosaccharide catalysis. J. Biol. Chem. 288, 14624–14635. 10.1074/jbc.M113.459438 23558681 PMC3656314

[B16] Dawood A. Ma K. (2020). Applications of microbial β-mannanases. Front. Bioeng. Biotechnol. 8, 598630. 10.3389/fbioe.2020.598630 33384989 PMC7770148

[B17] Dhawan S. Kaur J. (2007). Microbial mannanases: an overview of production and applications. Crit. Rev. Biotechnol. 27, 197–216. 10.1080/07388550701775919 18085462

[B18] Do B. C. Dang T. T. Berrin J. G. Haltrich D. To K. A. Sigoillot J. C.. (2009). Cloning, expression in *Pichia. pastoris*, and characterization of a thermostable GH5 mannan endo-1,4-β-mannosidase from *Aspergillus niger* BK01. Microb. Cell. Fact. 8, 59. 10.1186/1475-2859-8-59 19912637 PMC2780388

[B19] Endo A. Nakamura S. Konishi K. Nakagawa J. Tochio T. (2016). Variations in prebiotic oligosaccharide fermentation by intestinal lactic acid bacteria. Int. J. Food. Sci. Nutr. 67, 125–132. 10.3109/09637486.2016.1147019 26888650

[B20] Eneyskaya E. V. Sundqvist G. Golubev A. M. Ibatullin F. M. Ivanen D. R. Shabalin K. A.. (2009). Transglycosylating and hydrolytic activities of the beta-mannosidase from *Trichoderma reesei*. Biochimie. 91, 632–638. 10.1016/j.biochi.2009.03.009 19327384

[B21] Ferreira H. M. Filho E. X. F. (2004). Purification and characterization of a β-mannanase from *Trichoderma. harzianum* strain T4. Carbohydr. Polym. 57, 23–29. 10.1016/j.carbpol.2004.02.010

[B22] Ge F. Zhu L. Aang A. Song P. Li W. Tao Y.. (2018). Recent advances in enhanced enzyme activity, thermostability and secretion by N-glycosylation regulation in yeast. Biotechnol. Lett. 40, 847–854. 10.1007/s10529-018-2526-3 29450673

[B23] Ghosh A. Luís A. S. Brás J. L. Fontes C. M. Goyal A. (2013). Thermostable recombinant β-(1 → 4)-mannanase from *C. thermocellum*: biochemical characterization and manno-oligosaccharides production. J. Agric. Food. Chem. 61, 12333–12344. 10.1021/jf403111g 24224831

[B24] Gómez B. Míguez B. Yáñez R. Alonso J. L. (2017). Manufacture and properties of glucomannans and glucomannooligosaccharides derived from konjac and other sources. J. Agric. Food. Chem. 65, 2019–2031. 10.1021/acs.jafc.6b05409 28248105

[B25] Goyal D. Kumar K. Centeno M. Thakur A. Pires V. Bule P.. (2019). Molecular cloning, expression and biochemical characterization of a family 5 glycoside hydrolase first endo-mannanase (RfGH5_7) from *Ruminococcus. flavefaciens* FD-1 v3. Mol. Biotechnol. 61, 826–835. 10.1007/s12033-019-00205-2 31435842

[B26] Han M. Wang X. Ding H. Jin M. Yu L. Wang J.. (2014). The role of N-glycosylation sites in the activity, stability, and expression of the recombinant elastase expressed by *Pichia pastoris*. Enzyme. Microb. Technol. 54, 32–37. 10.1016/j.enzmictec.2013.09.014 24267565

[B27] Hlalukana N. Magengelele M. Malgas S. Pletschke B. I. (2021). Enzymatic conversion of mannan-rich plant waste biomass into prebiotic mannooligosaccharides. Foods. 10, 2010. 10.3390/foods10092010 34574120 PMC8468410

[B28] Hoshida H. Fujita T. (2013). Cha-aim K, Akada R. N-Glycosylation deficiency enhanced heterologous production of a *Bacillus licheniformis* thermostable alpha-amylase in *Saccharomyces cerevisiae*. Appl. Microbiol. Biotechnol. 97, 5473–5482. 10.1007/s00253-012-4582-2 23306636

[B29] Jana U. K. Kango N. (2020). Characteristics and bioactive properties of mannooligosaccharides derived from agro-waste mannans. Int. J. Biol. Macromol. 149, 931–940. 10.1016/j.ijbiomac.2020.01.304 32014482

[B30] Jana U. K. Suryawanshi R. K. Prajapati B. P. Kango N. (2021). Prebiotic mannooligosaccharides: synthesis, characterization and bioactive properties. Food. Chem. 342, 128328. 10.1016/j.foodchem.2020.128328 33257024

[B31] Jana U. K. Suryawanshi R. K. Prajapati B. P. Soni H. Kango N. (2018). Production optimization and characterization of mannooligosaccharide generating β-mannanase from *Aspergillus oryzae*. Bioresour. Technol. 268, 308–314. 10.1016/j.biortech.2018.07.143 30092484

[B32] Jiang Z. Wei Y. Li D. Li L. Chai P. Kusakabe I.. (2006). High-level production, purification and characterization of a thermostable β-mannanase from the newly isolated *Bacillus. subtilis* WY34. Carbohydr. Polym. 66, 88–96. 10.1016/j.carbpol.2006.02.030

[B33] Kaira G. S. Kapoor M. (2021). Molecular advancements on over-expression, stability and catalytic aspects of endo-β-mannanases. Crit. Rev. Biotechnol. 41, 1–15. 10.1080/07388551.2020.1825320 33032458

[B34] Karbalaei M. Rezaee S. A. Farsiani H. (2020). *Pichia. pastoris*: a highly successful expression system for optimal synthesis of heterologous proteins. J. Cell. Physiol. 235, 5867–5881. 10.1002/jcp.29583 32057111 PMC7228273

[B35] Katrolia P. Yan Q. Zhang P. Zhou P. Yang S. Jiang Z.. (2013). Gene cloning and enzymatic characterization of an alkali-tolerant endo-1,4-β-mannanase from *Rhizomucor miehei*. J. Agric. Food. Chem. 61, 394–401. 10.1021/jf303319h 23252695

[B36] Katrolia P. Zhou P. Zhang P. Yan Q. Li Y. Jiang Z.. (2012). High level expression of a novel β-mannanase from *Chaetomium* sp. exhibiting efficient mannan hydrolysis. Carbohydr. Polym. 87, 480–490. 10.1016/j.carbpol.2011.08.008 34662993

[B37] Kim D. Y. Ham S. J. Lee H. J. Kim Y. J. Shin D. H. Rhee Y. H.. (2011). A highly active endo-β-1,4-mannanase produced by *Cellulosimicrobium* sp. strain HY-13, a hemicellulolytic bacterium in the gut of *Eisenia fetida*. Enzyme. Microb. Technol. 48, 365–370. 10.1016/j.enzmictec.2010.12.013 22112951

[B38] Kumar Suryawanshi R. Kango N. (2021). Production of mannooligosaccharides from various mannans and evaluation of their prebiotic potential. Food. Chem. 334, 127428. 10.1016/j.foodchem.2020.127428 32688173

[B39] Li L. Qu M. Liu C. Pan K. Xu L. (2019). OuYang K, et al. Expression of a recombinant *Lentinula edodes*. cellobiohydrolase by *Pichia. pastoris* and its effects on in vitro ruminal fermentation of agricultural straws. Int. J. Biol. Macromol. 134, 146–155. 10.1016/j.ijbiomac.2019.05.043 31077694

[B40] Li Y. X. Yi P. Liu J. Yan Q. J. Jiang Z. Q. (2018). High-level expression of an engineered β-mannanase (mRmMan5A) in *Pichia. pastoris* for manno-oligosaccharide production using steam explosion pretreated palm kernel cake. Bioresour. Technol. 256, 30–37. 10.1016/j.biortech.2018.01.138 29428611

[B41] Liao H. Li S. Zheng H. Wei Z. Liu D. Raza W.. (2014). A new acidophilic thermostable endo-1,4-β-mannanase from *Penicillium. oxalicum* GZ-2: cloning, characterization and functional expression in. *Pichia. pastoris*. BMC. Biotechnol. 14, 90. 10.1186/s12896-014-0090-z 25348022 PMC4219100

[B42] Liu Z. Ning C. Yuan M. Yang S. Wei X. Xiao M.. (2020). High-level expression of a thermophilic and acidophilic β-mannanase from *Aspergillus kawachii*. IFO 4308 with significant potential in mannooligosaccharide preparation. Bioresour. Technol. 295, 122257. 10.1016/j.biortech.2019.122257 31648129

[B43] Looser V. Bruhlmann B. Bumbak F. Stenger C. Costa M. Camattari A.. (2015). Cultivation strategies to enhance productivity of *Pichia pastoris*: a review. Biotechnol. Adv. 33, 1177–1193. 10.1016/j.biotechadv.2015.05.008 26027890

[B44] Ma L. Ma Q. Cai R. Zong Z. Du L. Guo G.. (2018). Effect of β-mannanase domain from *Trichoderma. reesei* on its biochemical characters and synergistic hydrolysis of sugarcane bagasse. J. Sci. Food. Agric. 98, 2540–2547. 10.1002/jsfa.8741 29028116

[B45] Malgas S. van Dyk S. J. Pletschke B. I. (2015). β-mannanase (Man26A) and α-galactosidase (Aga27A) synergism - a key factor for the hydrolysis of galactomannan substrates. Enzyme. Microb. Technol. 70, 1–8. 10.1016/j.enzmictec.2014.12.007 25659626

[B46] Mao Y. H. Song A. X. Yao Z. P. Wu J. Y. (2018). Protective effects of natural and partially degraded konjac glucomannan on *Bifidobacteria* against antibiotic damage. Carbohydr. Polym. 181, 368–375. 10.1016/j.carbpol.2017.10.083 29253985

[B47] Miller G. L. (1959). Use of dinitrosalicylic acid reagent for determination of reducing sugar. Anal. Biochem. 31, 426–428. 10.1021/ac60147a03033274222

[B48] Moreira L. R. Filho E. X. (2008). An overview of mannan structure and mannan-degrading enzyme systems. Appl. Microbiol. Biotechnol. 79, 165–178. 10.1007/s00253-008-1423-4 18385995

[B49] Pham T. A. Berrin J. G. Record E. To K. A. Sigoillot J. C. (2010). Hydrolysis of softwood by *Aspergillus* mannanase: role of a carbohydrate-binding module. J. Biotechnol. 148, 163–170. 10.1016/j.jbiotec.2010.05.012 20541570

[B50] Pongsapipatana N. Damrongteerapap P. Chantorn S. Sintuprapa W. Keawsompong S. Nitisinprasert S.. (2016). Molecular cloning of kman coding for mannanase from *Klebsiella oxytoca* KUB-CW2-3 and its hybrid mannanase characters. Enzyme. Microb. Technol. 89, 39–51. 10.1016/j.enzmictec.2016.03.005 27233126

[B51] Ponzini E. Natalello A. Usai F. Bechmann M. Peri F. Müller N.. (2019). Structural characterization of aerogels derived from enzymatically oxidized galactomannans of fenugreek, sesbania and guar gums. Carbohydr. Polym. 207, 510–520. 10.1016/j.carbpol.2018.11.100 30600034

[B52] Rosengren A. Reddy S. K. Sjöberg J. S. Aurelius O. Logan D. T. Kolenov,á K.. (2014). An *Aspergillus nidulans* β-mannanase with high transglycosylation capacity revealed through comparative studies within glycosidase family 5. Appl. Microbiol. Biotechnol. 98, 10091–10104. 10.1007/s00253-014-5871-8 24950755 PMC4237917

[B53] Rungruangsaphakun J. Keawsompong S. (2018). Optimization of hydrolysis conditions for the mannooligosaccharides copra meal hydrolysate production. Biotech. 8, 169. 10.1007/s13205-018-1178-2 29527456 PMC5843564

[B54] Sabini E. Schubert H. Murshudov G. Wilson K. S. Siika-Aho M. Penttil,ä M.. (2000). The three-dimensional structure of a *Trichoderma. reesei* β-mannanase from glycoside hydrolase family. Acta. Crystallogr. D. Biol. Crystallogr. 56, 3–13. 10.1107/S0907444999013943 10666621

[B55] Sébastien G. Christophe B. Mario A. Pascal L. Michel P. Aurore R.. (2014). Impact of purification and fractionation process on the chemical structure and physical properties of locust bean gum. Carbohydr. Polym. 108, 159–168. 10.1016/j.carbpol.2014.02.092 24751260

[B56] Sharma P. Sharma S. Ramakrishna G. Srivastava H. Gaikwad K. A. (2022). Comprehensive review on leguminous galactomannans: structural analysis, functional properties, biosynthesis process and industrial applications. Crit. Rev. Food. Sci. Nutr. 62, 443–465. 10.1080/10408398.2020.1819196 33012173

[B57] Singh S. Singh G. Arya S. K. (2018). Mannans: an overview of properties and application in food products. Int. J. Biol. Macromol. 119, 79–95. 10.1016/j.ijbiomac.2018.07.130 30048723

[B58] Song Y. Sun W. Fan Y. Xue Y. Liu D. Ma C.. (2018). Galactomannan degrading enzymes from the mannan utilization gene cluster of alkaliphilic *Bacillus* sp. N16-5 and their synergy on galactomannan degradation. J. Agric. Food. Chem. 66, 11055–11063. 10.1021/acs.jafc.8b03878 30351049

[B59] Srivastava P. K. Kapoor M. (2017). Production, properties, and applications of endo-β-mannanases. Biotechnol. Adv. 35, 1–19. 10.1016/j.biotechadv.2016.11.001 27836790

[B60] Srivastava P. K. Panwar D. Prashanth K. V. Kapoor M. Structural characterization in vitro fermentation of β-mannooligosaccharides produced from locust bean gum by GH-26 endo-β-1, 4.-mannanase (ManB-1601). J Agric Food Chem. (2017) 65:2827–38. 10.1021/acs.jafc.7b00123 28225615

[B61] Sun D. Zhang J. Li C. Wang T. F. Qin H. M. (2021). Biochemical and structural characterization of a novel thermophilic and acidophilic β-mannanase from *Aspergillus calidoustus*. Enzyme. Microb. Technol. 150, 109891. 10.1016/j.enzmictec.2021.109891 34489044

[B62] Taylor I. I. L. E. Knott B. C. Baker J. O. Alahuhta P. M. Hobdey S. E. Linger J. G.. (2018). Engineering enhanced cellobiohydrolase activity. Nat. Commun. 9, 1186. 10.1038/s41467-018-03501-8 29567941 PMC5864845

[B63] Wang H. Luo H. Li J. Bai Y. Huang H. Shi P.. (2010). An α-galactosidase from an acidophilic *Bispora* sp. MEY-1 strain acts synergistically with β-mannanase. Bioresour. Technol. 101, 8376–8382. 10.1016/j.biortech.2010.06.045 20591661

[B64] Xia W. Lu H. Xia M. Cui Y. Bai Y. Qian L.. (2016). A novel glycoside hydrolase family 113 endo-β-1,4-mannanase from *Alicyclobacillus* sp. strain A4 and insight into the substrate recognition and catalytic mechanism of this family. Appl. Environ. Microbiol. 82, 2718–2727. 10.1128/AEM.04071-15 26921423 PMC4836435

[B65] Xie J. Wang B. He Z. Pan L. A. (2020). thermophilic fungal GH36 α-galactosidase from *Lichtheimia. ramosa* and its synergistic hydrolysis of locust bean gum. Carbohydr. Res. 491, 107911. 10.1016/j.carres.2020.107911 32217360

[B66] Yamabhai M. Sak-Ubol S. Srila W. Haltrich D. (2016). Haltrich, Mannan biotechnology: from biofuels to health. Crit. Rev. Biotechnol. 36, 32–42. 10.3109/07388551.2014.923372 25025271

[B67] Yang M. Yu X. W. Zheng H. Sha C. Zhao C. Qian M.. (2015). Role of N-linked glycosylation in the secretion and enzymatic properties of *Rhizopus chinensis* lipase expressed in *Pichia pastoris*. Microb. Cell. Fact. 14, 2–14. 10.1186/s12934-015-0225-5 25880561 PMC4417512

[B68] Yang Z. Zhang Z. (2018). Engineering strategies for enhanced production of protein and bio-products in *Pichia. pastoris*: a review. Biotechnol. Adv. 36, 182–195. 10.1016/j.biotechadv.2017.11.002 29129652

[B69] Zhang R. Song Z. Wu Q. Zhou J. Li J. Mu Y.. (2016). A novel surfactant-, NaCl-, and protease-tolerant β-mannanase from *Bacillus* sp. HJ14. Folia. Microbiol. 61, 233–242. 10.1007/s12223-015-0430-y 26489953

[B70] Zheng F. Han T. Basit A. Liu J. Miao T. Jiang W.. (2022). Whole-genome sequence and comparative analysis of *Trichoderma. asperellum* ND-1 reveal its unique enzymatic system for efficient biomass degradation. Catalysts 12, 437. 10.3390/catal12040437

[B71] Zheng F. Liu J. Basit A. Miao T. Jiang W. (2018). Insight to improve α-L-arabinofuranosidase productivity in *Pichia. pastoris* and its application on corn stover degradation. Front. Microbiol. 9, 3016. 10.3389/fmicb.2018.03016 30631307 PMC6315152

[B72] Zheng F. Song L. Basit A. Liu J. Miao T. Wen J.. (2020). An endoxylanase rapidly hydrolyzes xylan into major product xylobiose via transglycosylation of xylose to xylotriose or xylotetraose. Carbohydr. Polym. 237, 116121. 10.1016/j.carbpol.2020.116121 32241400

[B73] Zhou J. Zhang R. Gao Y. Li J. Tang X. Mu Y.. (2012). Novel low-temperature-active, salt-tolerant and proteases-resistant endo-1,4-β- mannanase from a new *Sphingomonas* strain. J. Biosci. Bioeng. 113, 568–574. 10.1016/j.jbiosc.2011.12.011 22265897

