# Supplementary material for: Biochemical analyses of a novel acidophilic GH5 β-mannanase from Trichoderma asperellum ND-1 and its application in mannooligosaccharides production from galactomannans
Source: Front Microbiol. 2023 Jun 9;14:1191553. doi: 10.3389/fmicb.2023.1191553 (PMC10288326; doi:10.3389/fmicb.2023.1191553)
Supplement: Supplementary file 1 [file Data_Sheet_1.pdf]

Supporting Information for:

Biochemical analyses of a novel acidophilic GH5 β-mannanase from *Trichoderma asperellum* ND-1 and its application in mannooligosaccharides production from galactomannans

Authors: Fengzhen Zheng[1,*], Abdul Basit[2], Jiaqiang Wang[1], Huan Zhuang[3], Jun Chen[4], Jianfen Zhang[1]

Affiliations:
[1] College of Biological and Environmental Engineering, Zhejiang Shuren University, Hangzhou 310021, China.
[2] Department of Microbiology, University of Jhang, Jhang 35200, Pakistan.
[3] Department of ENT and Head & Neck Surgery, The Children's Hospital Zhejiang University School of Medicine, Zhejiang, Hangzhou, 310051, China.
[4] Interdisciplinary Research Academy, Zhejiang Shuren University, Hangzhou 310021, China.

***Corresponding author:** Fengzhen Zheng
E-mail address: 18811068358@163.com

Present address: College of Biological and Environmental Engineering, Zhejiang Shuren University, Hangzhou 310021, China.

Supplementary Table S1. Oligonucleotide primers used in this study.

Primers	Sequence (5′- 3′)
TaMan5-F	GCCTTGTTGGCTGCAACCT CGGCAG
TaMan5-R	TTAGCTGGGCACACACTGAGAGTAG
AOX-F	GACTGGTTCCAATTGACAAGC
AOX-R	GCAAATGGCATTCTGACATCC
**TaMan5 mutants**	
D152A-F	CTGGTCTGCTTATGGTGGTATTAACGCTTACGTTAATGCTTTTGGTGGTAACGCTA CTTCTTGG
D152A-R	GCGTTAATACCACCATAAGCAGACCAGTTGTTAACGAAGTTAATAATCAACTTCA AAC
E205A-F	GGGAATTGGCTAATGCACCAAGATGTAACGGTTGCTCCACCTCTGTTATCTGGAAC TGGG
E205A-R	CTTGGTGCATTAGCCAATTCCCAAGCAAAAATGGCAGTACTAGTAGTATAACGAG A
E241A-F	TGATGCGGGTTTGGGATTGGCTACTGGATCTGATGGTTCTTATCCTTACAC
E241A-R	CCCAAACCCGCATCACCCAAAGTGACCAAATGGTTTGGGTCCAGAGAC
E259A-F	CGGTGCAGGTACTGATTTTGCTTCTTACATGAACATTACTACTTTGG
E259A-R	CAAAATCAGTACCTGCACCGTAAGTGTAAGGATAAGAACCATCAGATCC
E313A-F	ATGTTGGAGGCGTACGGTGCTCCAACTAACCATTGTGCTATTGAATCACCATGGC
E313A-R	ACCGTACGCCTCCAACATGCATGGTTTACCAGAAGCAACACAAGC
D356A-F	CTTCTGCCGATGGAAACACTGTTTACTACGGTACTTCTGATTTTACTTGTTTGG
D356A-R	GTGTTTCCATCGGCAGAAGATTGACCGTTAGACAAAGTATCTCCCCATTGCC
D357A-F	CTTCTGACGCTGGAAACACTGTTTACTACGGTACTTCTGATTTTACTTGTTTGG
D357A-R	AGTGTTTCCAGCGTCAGAAGATTGACCGTTAGACAAAGTATCTCCCCATTGCC

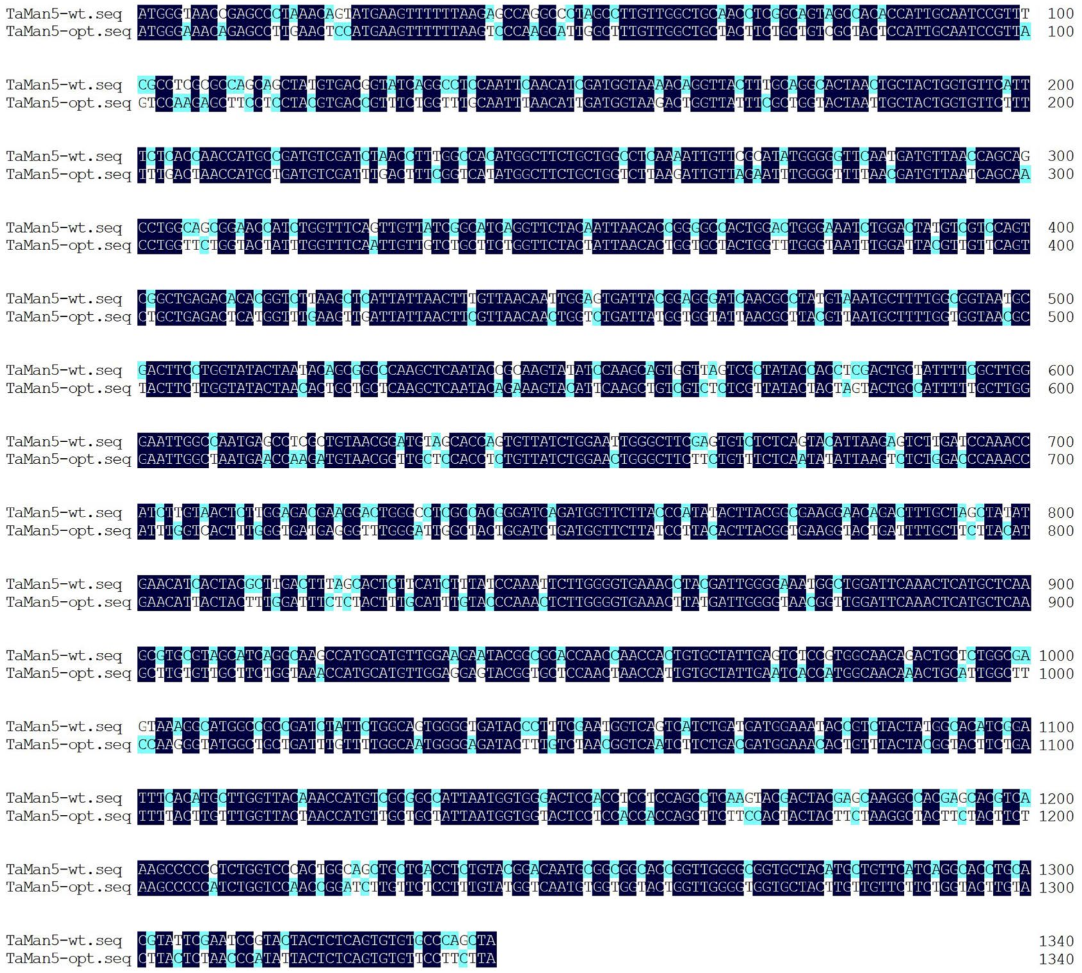

Supplementary Figure S1. Alignment of original β-mannanase gene (*TaMan5*-wt) sequence and optimized type gene (*TaMan5*-opt) sequence.

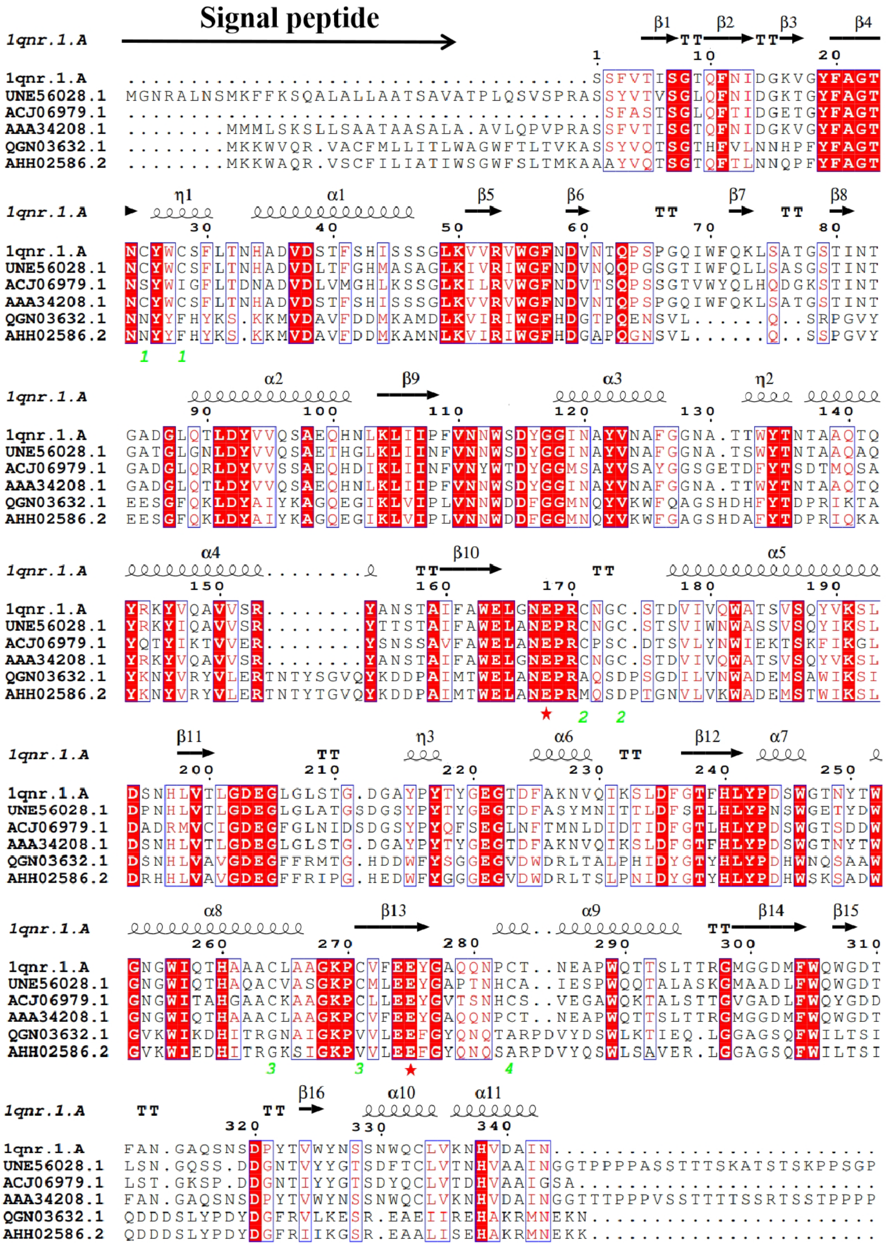

Supplementary Figure S2. Multiple sequence alignment of TaMan5 (GenBank accession No. UNE56028.1) with other GH 5 β-mannanases, including *Trichoderma reesei* (PDB ID code: 1qnr.1.A), *Aspergillus niger* BK01 (ACJ06979.1), *Bacillus* sp. KW1 (QGN03632.1) and *Bacillus* sp. HJ14 (AHH02586.2). The two catalytic residues (E205 and E313) of TaMan5 are marked using red asterisks. The alignment was carried out by ClustalX2 and ESPript 3.0.

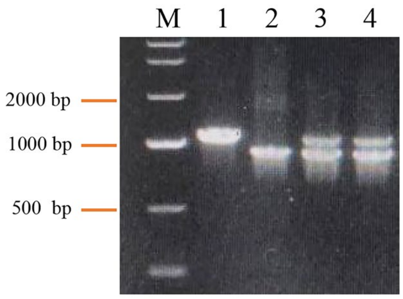

Supplementary Figure S3. Confirmation of engineered strains TaMan5-wt and TaMan5-opt using PCR primer AOX-F/AOX-R. Lanes: M, DNA markers; 1, negative control; 2, positive control; 3, recombinant strain TaMan5-wt; 4, recombinant strain TaMan5-opt.

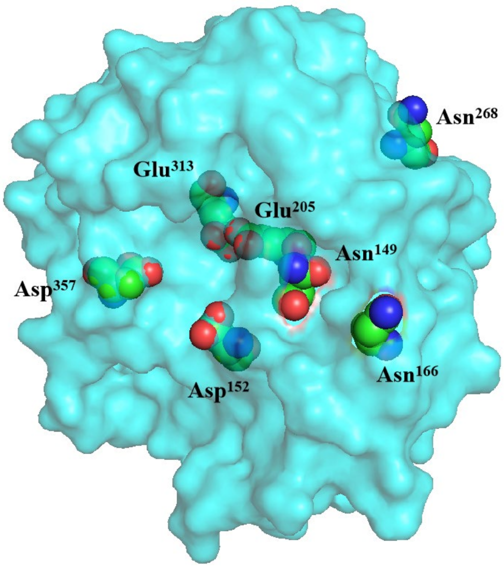

Supplementary Figure S4. 3D structure of TaMan5 simulated by SWISS-Model software. Predicted catalytic sites (Glu^{205}, Glu^{313}, Asp^{152} and Asp^{357}) and three N-glycosylation sites (Asn^{149}, Asn^{166} and Asn^{268}) of TaMan5 are marked, respectively.
